# Research on Passivation Simulation of Plasticizer N-Butylnitroxyethylnitramine (BuNENA) in Hydroxy-Terminated Polyether (HTPE) Propellants

**DOI:** 10.3390/polym17091147

**Published:** 2025-04-23

**Authors:** Zhiming Guo, Riccardo Rossi, Rui Deng, Yuheng Wu, Hanwen Liu, Lin Hao, Xiaolong Fu

**Affiliations:** 1 Departament d’Enginyeria Civil i Ambiental (DECA), Universitat Politécnica de Catalunya (UPC), Jordi Girona 1, 08034 Barcelona, Spain; zhiming.guo@upc.edu (Z.G.); riccardo.rossi@upc.edu (R.R.); 2 School of Mechatronics Engineering, North University of China, Taiyuan 030051, China; 3 Xi’an Modern Chemistry Research Institute, Xi’an 710065, China; 18223783718@163.com (R.D.); wyh915839863@163.com (Y.W.); 3018001675@tju.edu.cn (H.L.); 4 School of Chemical Engineering and Technology, Tianjin University, Tianjin 300350, China; haolin@tju.edu.cn

**Keywords:** HTPE propellants, BuNENA, passivation simulation, bubble

## Abstract

N-butylnitroxyethylnitramine (BuNENA) is a high-energy plasticizer with high plasticizing ability, low sensitivity, and high energy. It has broad application prospects in HTPE propellants. Nevertheless, as an energetic plasticizer, it requires treatment to reduce its sensitivity. To this end, the passivation process for BuNENA was simulated using a mixing model analogous to nucleate boiling. This method involves tracking the formation and movement of bubbles using a Lagrange frame, and the bubbles themselves are modeled as rigid spheres subject to buoyancy and viscous forces. A variational multiscale (VMS)-based Euler framework was employed to simulate the fluid surrounding the bubble. The movement process of the bubbles was analyzed, and it was found that the amount of bubbles and the movement speed were higher at high temperatures and in a high vacuum, and the passivation effect on BuNENA was better. At a pressure of 40 mbar and a temperature of 50 °C, BuNENA demonstrated an 89% water removal rate. A comparison of the experimental results with the simulation results revealed slight discrepancies between them. A meticulous analysis of the passivation process for BuNENA is rendered possible by integrating experimental and simulation methodologies, a feat that has immense implications for the realm of composite solid propellant passivation.

## 1. Introduction

Nitroxyethylnitramine compounds (NENAs) have the general formula RN(NO_2_)CH_2_CH_2_ONO_2_, where R is methyl, ethyl, propyl, isopropyl, butyl, and amyl. The majority of NENA plasticizers exhibit a high melting point and high volatility. However, N-butylnitroxyethylnitramine (BuNENA) is distinguished by its notably low melting point, minimal volatility, and glass transition temperature of −82 °C [1]. BuNENA features nitramine and nitrate energy groups, exhibiting excellent thermochemical properties, including high heat of formation, high density, and low average molecular weight of combustion products. Furthermore, it has been demonstrated to possess a high degree of plasticizing efficacy in nitrocellulose and other cellulose derivatives, as well as in polyether adhesives [2,3,4,5]. Compounds of BuNENA, which exhibit excellent physicochemical properties, have been utilized as energetic adhesives in a variety of solid propellants and explosive systems, including nitrate-plasticized polyether propellants (NEPEs), hydroxy-terminated polybutadiene propellants (HTPBs), high-energy polyether propellants (HTPEs), high-energy butyl polymer-bonded propellants and modified double-base propellants based on nitrocotton (CMDBs).

A significant amount of research has been conducted on BuNENA energetic plasticizers by researchers. Liao et al. [6] conducted thermogravimetric (TG) tests on a BuNENA-plasticized nitramine propellant, analyzed the thermal decomposition mechanism of the propellant using the model-fitting method, and predicted the storage life. The results showed that the storage life of the BuNENA-NC-RDX propellant (24.49 years) is significantly higher than that of the NG-NC-RDX propellant (16.33 years). Bayat et al. [7] utilized thermal and rheological analysis to examine the impact of diverse energetic plasticizers on the thermo-mechanical characteristics of polyurethane networks. The results demonstrated that, in comparison with the plasticizers of trihydroxyethane trinitrate (TMETN) and 1,2,4-butanediol trinitrate (BTTN), N-butylnitroxyethylnitramine (BuNENA) exhibited superior compatibility with the copolymers. Qi et al. [8] found that compared with NC/NG, the tensile strength and elongation at break measured by NC/BuNENA at −40 °C increased by about 23% and 33%, respectively, indicating that BuNENA has a higher plasticizing efficiency than NG. The results of jar electron microscopy (SEM) and meson dynamics simulation showed that the miscibility of NC and BuNENA is better than that of NG. Molecular dynamics simulation results showed that compared with the NC/NG mixture, NC/BuNENA exhibits stronger hydrogen bond and van der Waals force strength. Lu et al. [9] studied the thermal decomposition characteristics of BuNENA and NG and concluded that the activation energy (Ea) of BuNENA is 124.15 kJ/mol, and the pre-exponential factor (lnA) is 25.92 s^−1^; the activation energy (Ea) of NG is 108.01 kJ/mol, and the lnA is 22.16 s^−1^. It has been observed that the activation energy of BuNENA is higher than that of NG, which means that BuNENA has better thermal stability than NG. Kalapcrao et al. [10] found through research that BuNENA replaced DEP with a 5.97% mass fraction in AP/Al/RDX-modified double-base propellants, and the burning rate, energy, mechanical properties, and mechanical stability of the propellants were all improved. The burning rate of 7 MPa increased from 12.4 mm/s to 14.6 mm/s, but the pressure index of 5.0~7.0 MPa increased from 0.49 to 0.70. The explosion heat increased from 4536 kJ/kg to 4927 kJ/kg, representing a relative increase of 8.6%, and the theoretical combustion temperature remained essentially constant, with a marginal change of only 1 °C. Eva Landsem et al. [11] investigated the comprehensive properties of HMX-GAP-BuNENA-based propellants. The formulations (mass fraction) were as follows: 60% HMX, 21.7% GAP, and 9.54% BuNENA. The burning rate was 6.3 mm/s (6.9 MPa), 8.1 mm/s (10 MPa), the pressure index was 0.66, the theoretical specific impulse was 2154 N·s·kg^−1^, and the theoretical characteristic velocity was 1345 m/s. Yu et al. [12] investigated the mechanism of influence of BuNENA on the micro and macro properties of the PET/N-100 binder system. Quantum chemical calculation and MD simulation were carried out at the chemical bond and polymer chain levels. The solidification network structure, glass transition temperature and microphase separation of the plasticized binder reaction system were characterized with consistent results, and the mechanism of the plasticizer’s influence on the curing kinetics and mechanical properties of the binder system was proposed. Liu et al. [13] compared NG/BTTN and the insensitive energetic plasticizer BuNENA propellant by DSC testing and furnace combustion numerical simulation. In the discharge simulation, the BuNENA plasticizer demonstrated a lower response temperature and a smaller pre-response in the high-temperature region, and the response was milder than the NG/BTTN plasticizer propellant. Bhattacharjee et al. [14] studied the performance of an aluminum-free tetrazole-based grafted HTPB (Tetz-HTPB) propellant (AP/Tetz-HTPB/BuNENA/HMX), and the results showed that the use of Tetz-HTPB achieved a higher specific impulse ($I_{\mathrm{sp}}$) than the traditional HTPB-based formulation (268.6 s) and the density-specific impulse (${\rho I}_{\mathrm{sp}}$: 520.6 g/cm^3^·s) exceeded the traditional HTPB-based formula ($I_{\mathrm{sp}}$: 248.5 s; ${\rho I}_{\mathrm{sp}}$: 420.5 g/cm^3^·s; AP/HTPB/DOA).

In recent years, HTPE propellants using BuNENA have also been favored by researchers. Li et al. [15] conducted slow-drying tests on HTPE/BuNENA and HTPB/DOS propellants at the same energy level. The findings revealed that the response temperature of the HTPE/BuNENA propellant was 166 °C, the response type was combustion, and only the oven end cap was damaged, indicating a low response degree. Conversely, the HTPB propellants exhibited a reaction temperature of 210 °C, a reaction type of explosion, and a severely damaged baking chamber, indicating a higher degree of reaction. Luo et al. [16] investigated the correlation between the mechanical properties of an HTPE/BuNENA binder prepared in situ and the plasticization ratio (pl/po) within the range of 0.4~1.2. The thermal properties of the HTPE/BuNENA binder with pl/po 1.0 and the early stage of BuNENA evaporation were studied. The findings indicate that the pl/po ratio exerts a significant influence on the mechanical properties of the materials. It was established that when the pl/po ratio was 1.0, the mechanical properties of the HTPE/BuNENA binder prepared in situ were significantly enhanced in comparison with traditional HTPE propellants. Mohamed Abdullah et al. [17] reported the propellant of a cross-linked HTPE binder with N-butylnitroxyethylnitramine (BuNENA) as an energetic plasticizer and HP 4000D as an amorphous prepolymer plasticizer. It has been demonstrated to possess both a high specific impulse and favorable low-temperature mechanical properties.

The findings of the above research demonstrate that BuNENA has the capacity to reduce the hazard level and mechanical sensitivity of propellants, decrease the thermal hazard of propellants, and significantly enhance the mechanical properties of propellants, especially the low-temperature mechanical properties. Furthermore, its plasticizing effect on nitrocellulose is superior to that of NG. BuNENA’s low volatility compared to nitroglycerin (NG) and diethylene glycol dinitrate (DEGDN) allows for long-term storage of the propellant. However, BuNENA essentially has O−NO_2_ isomerization instability similar to nitrate ester plasticizers. The nitrogen oxides produced by decomposition not only affect the life of the propellant but also the combustion performance [18]. In order to ensure the performance of the propellant, the passivation treatment of BuNENA is essential.

In order to study the passivation process for BuNENA, a hybrid model similar to nucleate boiling was used to simulate the passivation process in the HTPE/BuNENA system. The optimal conditions for the passivation of BuNENA were established by monitoring the movement and size variations of bubbles during low-pressure boiling under various operational parameters. In two-phase bubbly flow simulations, the momentum exchange between phases is calculated via interfacial forces such as the drag, lift, turbulent dispersion, wall lubrication, and virtual mass. The most important force controlling the movement of the bubble is the resistance, which plays an obstructing role in the rise of the bubble and is usually represented by the dimensionless resistance coefficient $C_{d}$. The calculation of the resistance coefficient is the key to predicting the final speed of the bubble rise [19]. Therefore, accurate modeling of the drag force is crucial. Many drag models, such as the Schiller–Naumann, Ishii–Zuber, Tomiyama et al., Zhang–Vanderheyden, Grace et al., and Ma and Ahmadi models, have been extensively used to calculate the drag force in bubble column problems [20,21,22]. In existing resistance models, the Schiller and Naumann model predicts the axial gas and liquid velocities satisfactorily, but it underestimates the local void fraction at low superficial gas velocity [23,24], whereas the Ishii and Zuber model predicts the axial gas and liquid velocities well for low void fractions and superficial velocities, but it overestimates the void fraction [25]. The Tomiyama model generally provides good accuracy in terms of the void fraction for medium to high ReB and a low void fraction. However, when the complexity flow is present, the Tomiyama model overestimates the void fraction [26,27]. Studies are still needed for the Feng and Salibindla models [28]. It can be seen that the resistance model is still being developed and perfected.

In this study, the drag coefficient model proposed by Bo et al. [29] was utilized. In this model, the combination of the Weber number and Morton number can reasonably characterize the bubble shape deformation, while the combination of the Reynolds number, Eotvos number, and Morton number can reasonably characterize the resistance coefficient. Meanwhile, the passivation simulation utilizes an Eulerian framework based on the variational multiscale method (VMS) for the surrounding fluid. This multiscale approach captures the interaction between the bubbles and the fluid efficiently, particularly in cases where the bubbles are not fully resolved. In the VMS method, the large scales are resolved using an Eulerian framework, while the unresolved sub-grid scales are modeled using appropriate stabilization techniques, ensuring that both the bubble dynamics and the fluid flow are adequately captured, despite the coarse resolution. The VMS framework ensures numerical stability and accuracy across a range of scales, enabling effective simulation of the passivation process in engineering applications. The passivation process for BuNENA was studied by simulating the movement of bubbles during low-pressure micro-boiling at different vacuum degrees and temperatures.

## 2. Experiment Setup

### 2.1. Materials

High-risk raw material solutions are classified as Class III explosives and inherently carry certain risks. Since the formation of bubbles during the vacuum boiling process does not involve any chemical reactions, alternative materials were selected for use in this experiment. In this paper, the substitute material used was prepared by dissolving glycerol and BuNENA in certain proportions. Both of these substances exhibit high boiling points and possess excellent thermal stability. They are miscible with water and also demonstrate mutual miscibility with one another. The viscosity of the high-hazard raw material solution is comparable to that of HTPE. The viscosities of the alternative materials and HTPE were determined at room temperature and 50 °C by means of a viscometer (HAKKE RS300 rotational rheometer, Thermo Fisher Scientific, Inc., Waltham, MA, USA). The results are presented in Table 1. It was found that when the volume ratio of glycerol to BuNENA was 11:8, the viscosity of the substitute material was essentially equivalent to that of HTPE. Glycerol, BuNENA, and HTPE were all provided by the Xi’an Institute of Modern Chemistry (Xi’an, China).

### 2.2. Substitute Material Preparation

A total of 500 milliliters of HTPE was transferred into a 500-milliliter beaker. The viscosity of the HTPE was subsequently measured at both room temperature and 50 °C using a viscometer. Glycerol and BuNENA were thoroughly mixed in an appropriate ratio, after which 500 milliliters of the resulting solution were poured into another 500-milliliter beaker. Following this, the viscosity of the mixed solution was measured at both room temperature and 50 °C with the same viscometer. A 50-milliliter aliquot of the mixed solution was then weighed using an electronic balance, and its mass was recorded at ambient temperature. The density of the mixed solution was calculated and is documented in Table 1.

### 2.3. Passivation Experiment

**Figure 1:**
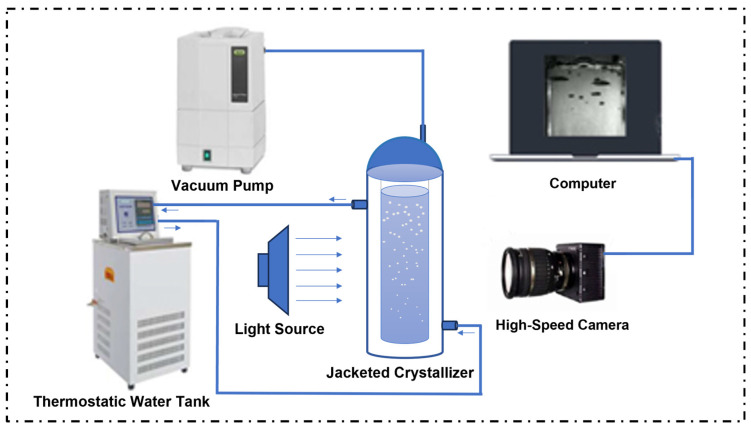
Passivation experiment system.

As illustrated in Figure 1, the passivation experiment system comprises a jacketed crystallizer (Suzhou Zhiyue Glassware Co., Ltd., located in Suzhou, China), a vacuum pump (VSV-20P, Zhejiang Feiyue Electromechanical Co., Ltd., located in Taizhou City, China), a circulating thermostatic water tank (Hangzhou Qiwei Instrument Co., Ltd., located in Hangzhou, China), a high-speed camera (Thousand-eyed Wolf X190, Hefei Zhongke Junda Vision Technology Co., Ltd., located in Hefei, China), and a computer. The reduced pressure micro-boiling process is carried out in the mold. Utilizing a high-speed camera at varying temperatures and vacuum degrees, the boiling process is recorded at a frame rate of 100. The high-speed camera comprises a variety of components, including optical imaging, signal transmission, control, image storage and processing, and other key components. These components work together to convert the target image into an electrical signal through optical imaging. Subsequent to the signal processing and transmission, the computer reads, displays, and analyzes the image to achieve fast, multiple sampling and accurate capture of high-speed targets.

The prepared materials were added to the crystallizer. Firstly, the target temperature of the constant temperature bath was adjusted; once the thermometer reached and maintained the set temperature for a period of time, it was removed. Then, the vacuum pump was connected and the desired vacuum level was set. After achieving and stabilizing this value, preparations were made for filming. Subsequently, the boiling conditions were recorded at a frame rate of 100 fps, and the operations were repeated after adjusting the parameters as necessary. Finally, the vacuum pump and constant temperature bath were turned off to allow cooling, after which the waste liquid was discharged, the equipment was disassembled and cleaned thoroughly, and the experiment was concluded. The experimental temperature and vacuum degree parameters are shown in Table 2.

### 2.4. Determination of Moisture Content and Video Recognition Test

In order to determine the effect of the passivation experiment, the water content of the BuNENA samples before and after the passivation experiment was determined by the Carl Fischer method.

According to the selected bubble movement model, the video of the bubble movement under reduced pressure and micro-boiling was simulated and analyzed on the computer [30]. The model described was implemented on top of Kratos V10.0, leveraging the Python 3.12.3 API in the implementation. Vectorized access to the search algorithm was added to the code as part of the current project. The use of the NumPy API allowed all of the operations implemented to be performed in the user space (that is, from the Python scripting layer) without any loss of efficiency. The simulation algorithm was based on the following steps. 1. Optionally use an adaptive time step to accurately capture the motion of the bubbles. 2. Interpolate the fluid velocity, pressure, and temperature at the position of the bubbles. 3. Compute $T_{boil}$ at each bubble, solving the relevant nonlinear equation. 4. Compute the relevant drag coefficient on each bubble. 5. Compute the $F_{bub}=F_{b}+F_{d}$. 6. Update the location of the bubble center by the adaptive piecewise Runge–Kutta method, and solve the corresponding partial differential equation. 7. Apply $F_{d}$ to the fluid domain and solve the fluid domain. 8. Calculate the terminal velocity and boiling pressure, and post-process the results.

## 3. Theoretical Models

The model employed for the bubble motion process is analogous to that delineated in Reference [31], wherein the bubble is subjected to the buoyancy force $F_{b}$ and the drag force $F_{d}$.

### 3.1. Buoyancy Force

Due to the difference in density between the bubble and the fluid it displaces, the buoyancy force $F_{b}$ can be given by the weight difference, and its magnitude depends on the diameter of the bubble, as shown in Equation (1):

(1) $$F_{b}=V_{b}\cdot \left(\rho_{f}-\rho_{b}\right)\cdot g,$$

where $F_{b}$ is the buoyancy force, $V_{b}$ is the volume of the bubble, $\rho_{f}$ is the fluid density, $\rho_{b}$ is the bubble density, and g is the gravitational acceleration.

### 3.2. Drag Force

Bubbles rising in a fluid usually start out spherical when their radius is “small” then become elliptical and finally semispherical. From a computational point of view, it is still convenient to describe bubbles in terms of an equivalent radius *R* defined as the radius of the bubble with the same volume.

The motion and shape of such equivalent bubbles can then be characterized in terms of three dimensionless numbers:

(2) $$Re\;∶=\frac{2\rho_{f}∥v_{f}-v_{b}∥R}{\mu_{f}}\left(Reynolds\;Number\right),$$

(3) $$Eo\;∶=\frac{\left(\rho_{f}-\rho_{b}\right)∥g∥}{\sigma {\left(2R\right)}^{2}}\left(Eotv\ddot{o}s\;number\right),$$

(4) $$Mo\;∶=\frac{∥g∥\mu_{f}^{4}}{\sigma^{3}\mu_{f}}\left(Morton\;number\right),$$

The drag force is then computed using the following formulas:

(5) $$F_{d}=\left\{\begin{array}{c} -6\pi \mu R\left(v_{f}-v_{b}\right),\;\;Re_{b}<10^{-3}\left(Stokes\;regime\right)\\ -\frac{1}{2}C_{d}\rho_{f}A_{b}∥v_{f}-v_{b}∥\left(v_{f}-v_{b}\right),\;\;Re_{b}>10^{-3}\left(Stokes\;regime\right) \end{array}\right.,$$

where $F_{d}$ is the drag force, *µ* is the dynamic viscosity of the fluid, R is the radius of the bubble, $v_{f}$ is the velocity of the fluid, $v_{b}$ is the velocity of the bubble, $C_{d}$ is the drag coefficient, and $A_{b}\;∶=\pi R^{2}$ is the cross-sectional area of the bubble.

This force represents the mechanism by which a disturbance in the fluid flow exerts a force on the bubble. It is also the mechanism by which the bubbles drive the fluid motion.

The practical application of this formula depends on the calculation of the drag coefficient $C_{d}\;$. Such a coefficient is heavily influenced by the actual shape of the bubble (spherical, elliptic or semispherical), something that is taken into account in terms of the defining dimensionless numbers. A recent and in-depth study of the subject can be found in Reference [29]. In this study, the empirical formulas outlined in the aforementioned paper are employed:

(6) $$C_{d}=\sqrt{C_{d,Mei}^{2}-C_{d,Eo}^{2}},$$

(7) $$C_{d,,Mei}=\frac{16}{Re}\left\{1+{\left[\frac{8}{Re}+\frac{1}{2}\left(1+\frac{3.315}{Re^{1/2}}\right)\right]}^{-1}\right\},$$

(8) $$C_{aux_{1}}=10^{-1.23\log\left(Eo\right)+0.37\log\left(Mo\right)+1.6},$$

(9) $$C_{aux_{2}}=\frac{4Eo}{Eo+9.5},$$

(10) $$C_{aux_{3}}=\frac{8}{3}\;\frac{Eo}{0.876Eo+4.887},$$

(11) $$C_{d,Eo}=max\left(C_{aux_{1}},\min\left(C_{aux_{2}},C_{aux_{3}}\right)\right),$$

### 3.3. Boiling Condition and Bubble Growth

The onset of fluid boiling is characterized by the surpassing of a local threshold temperature, designated as the boiling temperature $T_{boil}$. As described in [32], the calculation of $T_{boil}$ as a function of the pressure necessitates the resolution of a nonlinear equation, a process that must be undertaken at every bubble position and for every time step. In the cases of interest, the atmospheric pressure is reduced by the creation of a vacuum so that the boiling threshold is reached in wide areas of the volume. This results in the formation of bubbles at “random” points within the volume (where disturbances are present in the real case). In the simulation process, this phenomenon must be replicated by prescribing nucleation points. In the present simulation, such nucleation points are generated at random positions within the domain, thereby achieving a user-defined bubble density. Bubbles are nucleated at such points with a small initial radius (in our simulation, we set $R_{0}=10^{-5}\;$ m).

Once nucleation has occurred, the subsequent growth of the bubbles is contingent on the quantity of gas produced by the phase change, which is in turn determined by the overheating $T-T_{boil}$.

In a preliminary phase of very short duration (Inertial range), the growth of the bubbles is retarded by the hydrodynamic resistance to their amplification. Here, the rate of change of the bubble radius R as it grows due to superheating is given by the following equation:

(12) $$\frac{dR}{dt}=\frac{2}{3}\cdot \frac{T-T_{boil}}{T_{boil}}\cdot \frac{L_{v}\rho_{b}}{\rho_{f}},$$

where $L_{v}$ is the latent heat of vaporization, $\rho_{b}$ is the bubble density, and $\rho_{f}$ is the fluid density.

In the second phase (which is of particular significance given the time frame under consideration in the present simulations), the expansion of the bubble radius is constrained by the heat transmission process at the bubble’s surface. A multitude of proposals (many of which are equivalent) have been advanced to model such growth [33]. The present study employs the relation described in [34]:

(13) $$R\;∶=\sqrt{\frac{12\alpha t}{\pi }}Ja,$$

where $Ja=\frac{\rho_{f}\rho_{c}\left(T-T_{boil}\right)}{\rho_{b}L_{v}}$ (Jackob number).

Most interestingly, and even if this does not represent the focus of the current work, the same approach could be employed in the simulation of pool boiling (when boiling happens on a surface). In this context, bubbles may also form at prescribed nucleation points, but they remain stable and do not grow in size unless a critical threshold is reached. This phenomenon is modeled by the introduction of a “nucleation condition”, which determines whether a specific nucleation site will ultimately become active. This is achieved by the following condition:

(14) $$T-T_{boil}>B_{pot},$$

(15) $$B_{pot}=\frac{2\sigma R_{gas}T_{boil}}{L_{v}P_{atm}d_{nuc}},$$

where *σ* is the surface tension, $R_{gas}$ is the universal gas constant, P is the pressure, and $d_{nuc}$ is the nucleation site diameter.

### 3.4. Fluid Model

The solver discretizes the incompressible Navier–Stokes equations described by the PDEs:

(16) $$\rho_{f}\frac{\partial u_{f}}{\partial t}+\rho u_{f}\cdot \nabla u_{f}-\nabla \cdot \tau +\nabla =\rho g+f_{d},$$

(17) $$\nabla \cdot u_{f}=0,$$

where $\rho_{f}$ is the density, $u_{f}$ represents the fluid velocity, p is the pressure and g is the gravity acceleration. The term $\tau \;\left(u_{f}\right)$ represents the shearing component of the Cauchy stress tensor, such that σ=τ+pI. As written, the model allows considering both Newtonian and non-Newtonian fluids by assuming that the following relation holds:

(18) $$\tau \;\;:=2\mu^{*}\nabla^{s}u,$$

Here, $\mu^{*}$ plays the role of a “secant” viscosity with an expression of the following type:

(19) $$\mu^{*}\;\;:=\left(k{\dot{\gamma }}^{n-1}+\frac{\tau_{y}}{\dot{\gamma }}\right),$$

with $\dot{\gamma \;}:=\sqrt{2\nabla^{s}u:\nabla^{s}u}$ and where k, n and τ_y_ are user-defined material parameters. Such a model cannot be implemented “as is” due to a singularity when $\dot{\gamma }$ = 0 [35]. A regularized version of the model entails the following expression:

(20) $$\mu^{*}\;\;:=\left(k{\dot{\gamma }}^{n-1}+\frac{\tau_{y}}{\dot{\gamma }}\left(1-e^{-m\dot{\gamma }}\right)\right),$$

where *m* is a user-defined parameter, which is thus employed in the implementation.

The discretization employs an equal order, the $u_{h}$ − $p_{h}$ formulation of simplicial technology. It is important to note that the discretization does not comply with the Ladyzhenskaya–Babuška–Brezzi (LBB) condition and therefore requires stabilization. The variational multiscale (VMS) method is employed here; a description of this method can be found in [36]. The essence of the VMS approach is to split the unknown in a resolved part, identified with a pedex “*h*”, and a sub-grid (unresolved) component identified with an apex as follows:

(21) $$u=u_{h}+u^{\prime },$$

(22) $$p=p_{h}+p^{\prime },$$

The sub-grid component is modeled in terms of the resolved residual at the Gauss point to give expressions of the following type:

(23) $$u^{\prime }\;\;:=\tau_{1}\Pi R_{\mathrm{mom}}\left(u_{h},p_{h}\right),$$

(24) $$p^{\prime }\;\;:=\tau_{2}\Pi R_{\mathrm{mass}}\left(u_{h},p_{h}\right),$$

where Π represents a projection operator, $R_{\mathrm{mom}\;}\left(u_{h},p_{h}\right)$ and $R_{\mathrm{mass}\;}$ are the residuals of the momentum and mass equations evaluated at the integration points of the mesh, and $\tau :=\frac{\rho }{\frac{1}{\delta t}+\frac{\mu^{*}}{h^{2}}+\frac{\rho ∥u_{h}∥}{h}}$. This splitting has been proven to be sufficient to stabilize both pressure instabilities and convection-related ones, while at the same time preserving the consistency of the formulation. Full details of the implemented formulation can be found in [36].

### 3.5. Model Verification

When the equivalent radius model used in this study is applied to water, the results are in good agreement with the values in the literature. When applied to glycerin (the material hypothesized in this experiment, for which, to our knowledge, no experimental results are available in the literature), the results obtained are markedly different from those obtained with still water. The specific model validation will be analyzed in Section 4.

## 4. Results and Discussion

### 4.1. Experimental Results of Video Recognition

**Figure 2:**
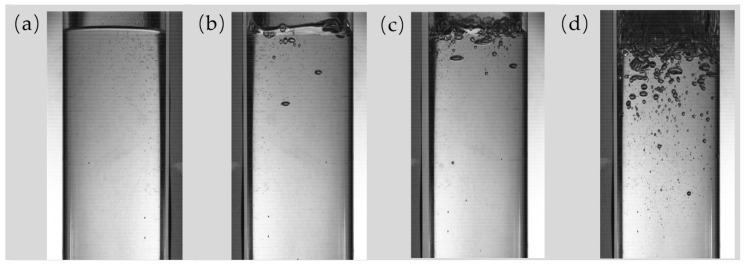
The boiling situation at 40 mbar and 50 °C: (**a**) almost no boiling; (**b**) mild boiling; (**c**) general boiling; and (**d**) violent boiling.

Under the working conditions of a vacuum pressure of 40 mbar and a temperature of 50 °C, the boiling behavior of the liquid is illustrated in Figure 2. When there is minimal boiling, the liquid level exhibits almost no fluctuation. With mild boiling, the liquid level fluctuates by approximately 4 mm. During general boiling, the fluctuations increase to around 14 mm. In the case of violent boiling, the liquid level fluctuates by approximately 26 mm.

**Figure 3:**
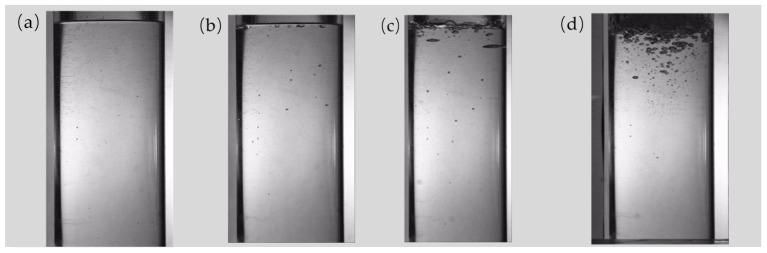
Experimental results under different working conditions: (**a**) vacuum 100 mbar, 70 °C; (**b**) vacuum 80 mbar, 64 °C; (**c**) vacuum 60 mbar, 58 °C; and (**d**) vacuum 40 mbar, 50 °C.

The experimental results obtained under varying working conditions are presented in Figure 3. It can be found that the number of bubbles is the largest when the working condition is 40 mbar and 50 °C. It is evident that as the pressure decreases, the number of bubbles shows a corresponding upward trend. This is because, as the pressure decreases, the boiling point of the liquid also decreases, enabling the interior of the liquid to more readily achieve saturated vapor pressure. Furthermore, the number of active gas nuclei increases. These factors collectively reduce the resistance encountered by bubbles during their movement, thereby facilitating both the formation and the growth of bubbles [37,38]. Conversely, an increase in pressure will result in a reduction in the liquid’s surface tension and an elevation in the steam density, which in turn influence the bubble nucleation, heat transfer efficiency, and critical heat flux during boiling [39,40].

**Figure 4:**
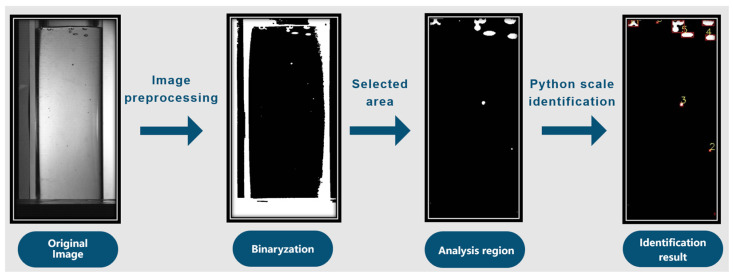
Bubble recognition process.

The bubble identification process is illustrated in Figure 4. Following the binarization of the boiling image, an analysis region is selected. Subsequently, utilizing Python, scale recognition is performed to obtain the parameters for the total area of bubble contour recognition and the number of bubbles at a specific temperature. The number of bubbles at a given moment is quantified using image recognition, and the total area of bubble contours is calculated by counting the pixels within each bubble and summing these values across all the bubbles. The bubble area identified through image recognition represents the cumulative area of all the pixel points enclosed within each bubble contour. In this study, the area of a single pixel point is approximately 0.419 mm^2^.

#### 4.1.1. Bubble Size and Parameters

**Figure 5:**
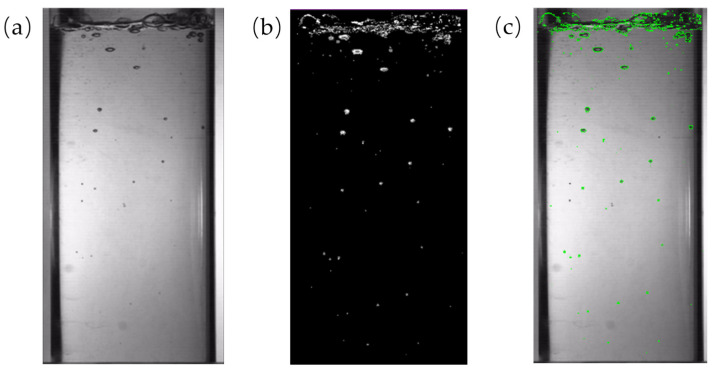
Bubble image identification result: (**a**) the original image; (**b**) the detected moving object; and (**c**) the recognized bubble.

**Figure 6:**
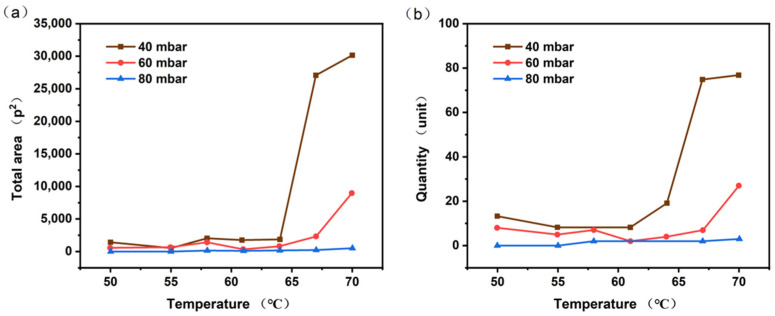
Bubble identification result (quantity and total area): (**a**) relationship between total bubble area and temperature; and (**b**) relationship between the total number of bubbles and temperature.

The results of the bubble identification process are presented in Figure 5 and Figure 6. As the temperature increases, both the number of bubbles and the total contour recognition area exhibit a consistent upward trend. This finding has been corroborated by a substantial body of research [41,42,43,44,45,46]. Notably, when the temperature rises from 64 °C to 67 °C, there is a pronounced increase in both the number of bubbles and the total contour recognition area. This is because the rise in temperature leads to an increase in the heat flux density and liquid supersaturation, as well as an enhancement in the density of the active nucleation sites. As a result, this gives rise to a higher frequency of bubble generation and a larger detachment diameter of bubbles [43,47,48]. Conversely, as the experimental pressure decreases, the number of bubbles and the total contour recognition area increase, reaching their peak values at 40 mbar and 70 °C (total area: 30,151.29589 p^2^; quantity: 76.80127 units). Based on the analysis of the total contour recognition area and the number of identified bubbles, it was concluded that the passivation experiment achieved more favorable outcomes under high-temperature and low-pressure conditions, effectively reducing the sensitivity of BuNENA.

#### 4.1.2. Bubble Velocity Analysis

The bubble that is readily discernible and susceptible to refraction is selected from the boiling image, and its path tracing is conducted to obtain the rising rate curve of the bubble movement. The highest point of the velocity curve is then designated as the analysis result. The specific calculation process is as follows. The centroid coordinates of the bubble are first calculated, and then the difference is found between the coordinates of the bubble in the current frame and the previous one. The result of this calculation is divided by the interval time between the two frames, and this is the current movement speed of the bubble. The following equation is used to express this calculation:

(25) $$v=\frac{\sqrt{{\left(P_{k,x}-P_{k-1,x}\right)}^{2}+{\left(P_{k,y}-P_{k-1,y}\right)}^{2}}}{\Delta t},$$

where $P_{k}$ is the center of mass position of the bubble at time *k*, $P_{k-1}$ is the center of mass position of the bubble at time *k* − 1, and Δt is the interval between two frames (known as 0.01 s).

It is important to note that, in theory, the movement direction of the bubble should be vertical and upward. However, due to the error in the installation of the equipment, the actual bubble exhibits a slight horizontal movement. Consequently, the speed of the bubble is defined as the moving distance of the bubble centroid between the two frames of data, rather than the vertical distance. In addition, the centroid position of the bubble is the pixel coordinate, necessitating offline calibration of the camera in advance to obtain the actual distance corresponding to a single pixel. In this study, the actual distance corresponding to a single pixel is determined to be 0.641 mm.

**Figure 7:**
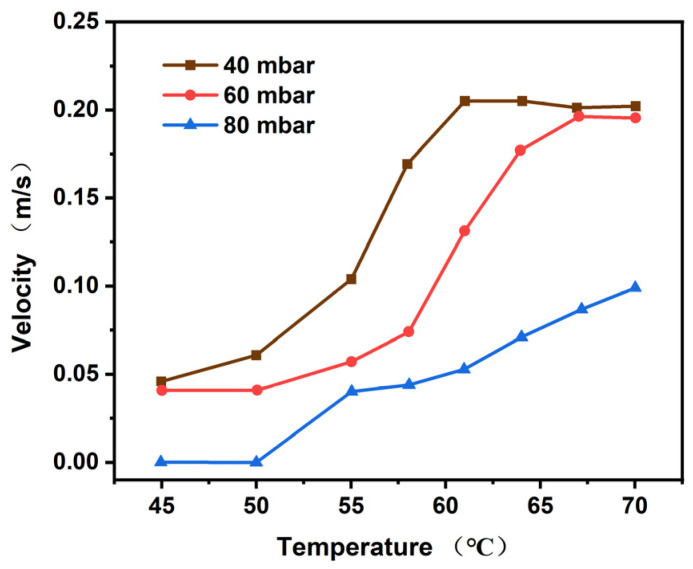
The velocity of the bubble varies with the temperature.

**Figure 8:**
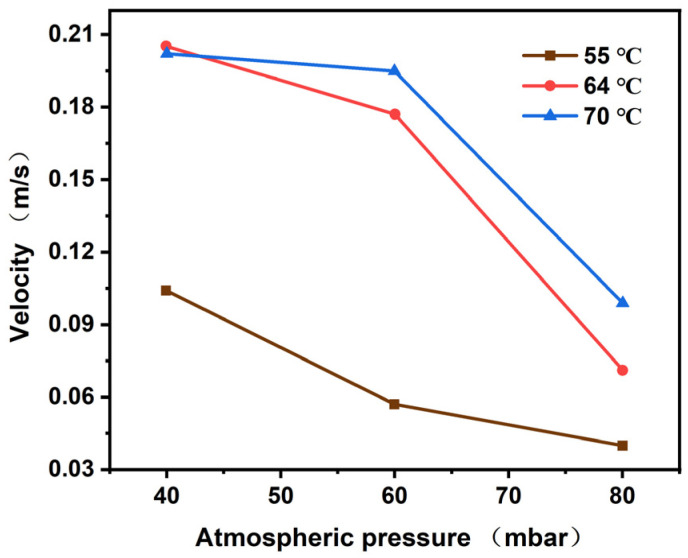
The velocity of the bubble varies with the atmospheric pressure.

As shown in Figure 7, the bubble velocity increases with the increase in temperature, and the movement velocity is at a maximum when the vacuum degree is 40 mbar. Figure 8 elucidates the relationship between the velocity of the bubble and the vacuum. It is evident from the data that as the temperature rises, the terminal speed of the bubble increases gradually. Furthermore, at lower pressures, the bubble terminal velocity is observed to be greater.

**Figure 9:**
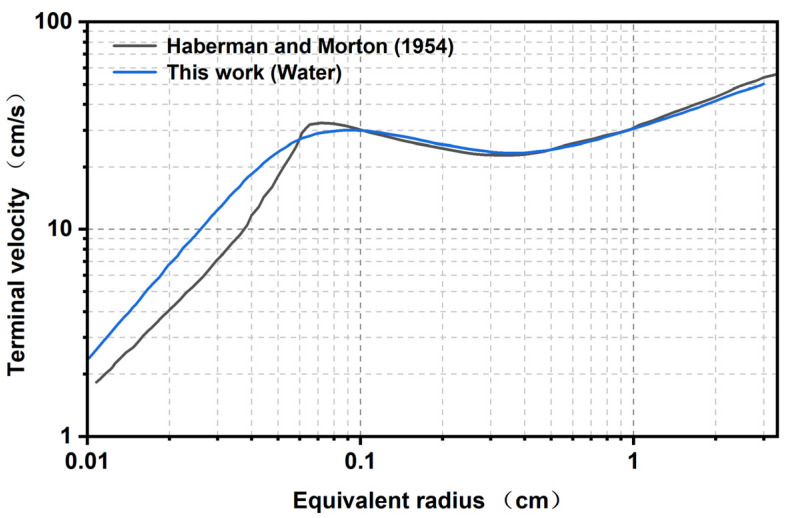
Distribution of the final velocities as a function of the equivalent radius in distilled water.

The equivalent radius model has been verified with the reference results for stationary water in [49]. The results demonstrate the terminal velocity achieved by a bubble with an equivalent radius of R, as shown in Figure 9, and closely correspond to the experimental values. It can be seen that the bubble motion model selected in this study has high accuracy and can be applied to simulate the movement of bubbles in the passivation process.

#### 4.1.3. Moisture Content Analysis

The primary objective of the passivation treatment of the energetic plasticizer BuNENA is the removal of moisture and the reduction of sensitivity. The moisture content of the BuNENA samples under the optimal condition (40 mbar, 50 °C) was determined. Prior to the initiation of the passivation experiment, the moisture content was recorded at 0.8%. Subsequent to the completion of the passivation experiment, the moisture content was determined to be 0.09%, and the water removal efficiency reached 89%.

### 4.2. Simulation Result

**Figure 10:**
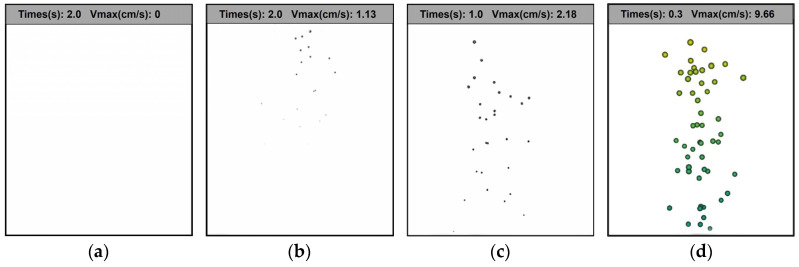
Images of the simulation results under different working conditions: (**a**) vacuum degree 100–70 °C; (**b**) vacuum degree 80–64 °C; (**c**) vacuum 60–58 °C; and (**d**) vacuum degree 40–50 °C.

The simulation results under different working conditions are shown in Figure 10. It is evident that the number and movement rate of the bubbles are significantly higher at 40 mbar than at 60 mbar, 80 mbar, and 100 mbar. This finding aligns with the experimental observations previously reported. When the vacuum degree is 40 mbar, the bubble appears earlier and the bubble moves faster. It can be seen that the passivation speed is faster under a high vacuum, and the water removal effect of BuNENA is better. The findings of the passivation simulation are in alignment with the experimental results, thereby substantiating the viability of the theoretical model of bubble movement and the passivation simulation method used in this study for elucidating the bubble movement during passivation.

### 4.3. Comparison of Experimental Results and Simulation Results

**Figure 11:**
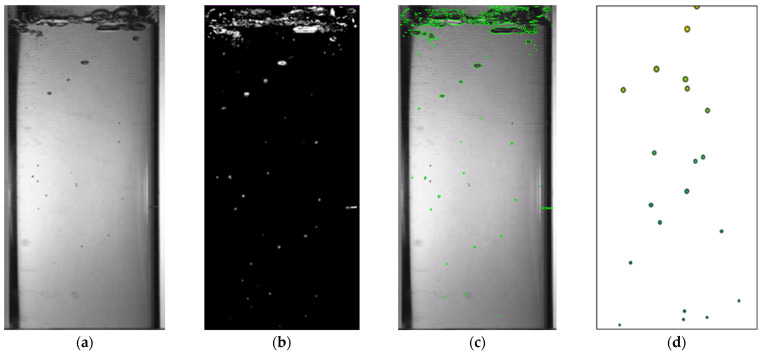
Comparison of the simulation results and image recognition results under the condition of vacuum 80 and 64 °C: (**a**) raw images; (**b**) detected moving objects; (**c**) identified bubbles; and (**d**) simulation results.

As illustrated in Figure 11, the dynamic behavior of the bubbles during the experiment exhibited significant variations in size and shape, which complicated the analysis process. Conversely, the movement of the bubbles rising after the simulation was more straightforward and discernible, facilitating a more straightforward analysis of the movement.

**Figure 12:**
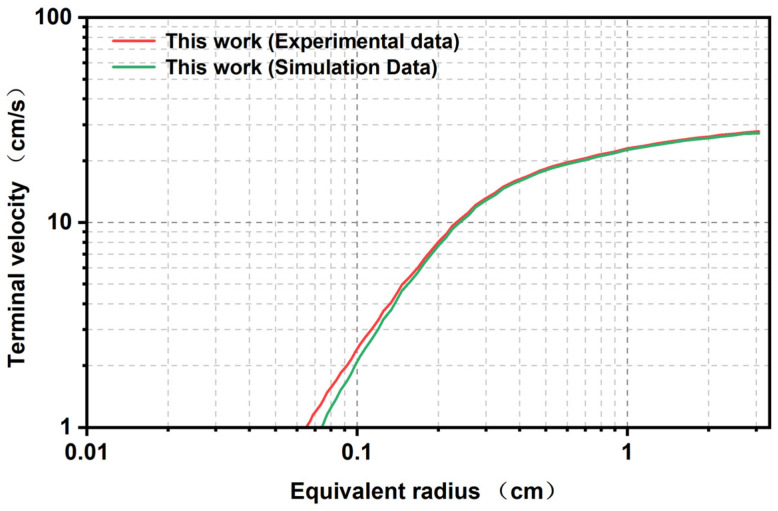
Distribution of the final velocity as a function of the equivalent radius: comparison of the experimental and simulation data.

The bubble movement model and simulation experiment method have been verified, and the accuracy of the bubble model and the feasibility of the simulation method have been confirmed. To further ascertain the precision of the simulation technique, the experimental data concerning the relationship between the bubble terminal rate and the equivalent radius were compared with the simulation data (Figure 12). As illustrated in the figure, following the transformation of the boiling substance into a glycerin + BuNENA sample, the terminal rate curve exhibits significant deviation from its water-based counterpart. However, it is evident that the simulation values exhibit a high degree of consistency with the experimental values. This observation serves as a solid validation of the efficacy of the passivation simulation method employed in this study.

## 5. Conclusions

This paper presents a comprehensive study of the passivation process for the energetic plasticizer BuNENA, integrating experimental and simulation methodologies. The analysis of the movement of bubbles in the passivation process for the BuNENA samples under different working conditions indicates that when the experimental temperature and vacuum degree increase, the amount of bubbles and the movement speed increase, which is conducive to the passivation reduction of BuNENA. At a pressure of 40 mbar and a temperature of 50 °C, the moisture content of the BuNENA samples decreased from 0.8% to 0.09%, exhibiting an 89% water removal rate. The selected bubble movement model was verified in water, and the experimental results showed only a slight discrepancy from the literature values. Utilizing these findings, a simulation was conducted, and the experimental outcomes exhibited strong concurrence with the simulation results. This outcome serves to substantiate the precision of the bubble motion model and the efficacy of the employed simulation methodology.

The present study demonstrates that the passivation process of an energetic plasticizer can be accurately analyzed through a combination of experiment and simulation. This finding is of great significance for the passivation process for composite solid propellants. It is not only conducive to exploring the best process conditions but also to improving the safety level of the passivation process.

## Figures and Tables

**Table 1:** Viscosity test results.

Materials	Viscosity (20 °C)/cP	Viscosity (50 °C)/cP	Density (20 °C) g/mL
HTPE	142.4	88	/
220 mL Glycerin + 160 mL BuNENA	148.8	81.6	1.17

**Table 2:** Experimental temperature and vacuum parameters (internal pressure).

Temperature/°C	45	50	55	58	61	64	67	70	75
Vacuum degree/mbar (Internal pressure/Pa)	40/60/80/100 4000/6000/8000/10,000								

## Data Availability

The data presented in this study are available on request from the corresponding author.
